# Gallbladder hemorrhage associated with segmental arterial mediolysis: a case report

**DOI:** 10.1186/s40792-023-01799-1

**Published:** 2024-01-08

**Authors:** Yuichi Hirose, Yusuke Tajima, Hiroki Sakata, Toshimasa Uekusa, Kentaro Kamada, Takashi Ikehara, Izuru Matsuda, Satomi Yoneyama, Akio Hidemura, Hiroyuki Suzuki

**Affiliations:** 1 Department of Surgery, Kanto Rosai Hospital, 1-1 Kizukisumiyoshicho, Nakahara-Ku, Kanagawa, 211-8510 Japan; 2 Department of Surgery, National Defense Medical College, 3-2 Namiki, Tokorozawa, Saitama 359-8513 Japan; 3 Department of Pathology, Kanto Rosai Hospital, 1-1 Kizukisumiyoshicho, Nakahara-Ku, Kanagawa, 211-8510 Japan; 4 Department of Gastrointestinal Medicine, Kanto Rosai Hospital, 1-1 Kizukisumiyoshicho, Nakahara-Ku, Kanagawa, 211-8510 Japan; 5 Department of Radiology, Kanto Rosai Hospital, 1-1 Kizukisumiyoshicho, Nakahara-Ku, Kanagawa, 211-8510 Japan

**Keywords:** Gallbladder hemorrhage, Segmental arterial mediolysis, Histopathology

## Abstract

**Background:**

Gallbladder hemorrhage is a rare but fatal condition. The reported causes of gallbladder hemorrhage include iatrogenesis, atherosclerotic changes in the cystic arteries, acute cholecystitis or cholelithiasis, malignancy, trauma, hemophilia, pseudoaneurysm, and the use of oral anticoagulant medications. Recently, segmental arterial mediolysis (SAM) has been reported as a possible etiology of life-threatening abdominal, retroperitoneal, and intracranial hemorrhages. However, no previous reports have described the association between gallbladder hemorrhage and SAM.

**Case presentation:**

A 59-year-old man was transferred to our hospital complaining of upper abdominal pain and vomiting. Contrast-enhanced computed tomography revealed high-density images of the gallbladder and common bile duct. However, there were no obvious findings of gallstones, cholecystitis, tumors, or aneurysms. He was diagnosed with gallbladder hemorrhage and bile duct obstruction. We performed a laparoscopic cholecystectomy after endoscopic biliary drainage. The gross appearance of the surgically resected specimen showed 12 small (3–12 mm), slightly elevated lesions on the gallbladder mucosa. Histologically, these slightly elevated lesions consisted of dilated muscular arteries of the gallbladder wall with fibrinoid degeneration of the media and focal loss of the internal and external elastic laminae. The histopathological diagnosis was confirmed as SAM.

**Conclusions:**

To the best of our knowledge, this is the first reported case of a gallbladder hemorrhage associated with SAM. Our case report shows that SAM can cause gallbladder hemorrhage, suggesting that SAM should be considered in the differential diagnosis of gallbladder hemorrhage.

## Background

Gallbladder hemorrhage is a rare but fatal condition [1–3]. Iatrogenesis, atherosclerotic changes in the cystic arteries, acute cholecystitis or cholelithiasis, malignancy, trauma, hemophilia, pseudoaneurysm, and the use of oral anticoagulant medications are reported as the causes of gall bladder hemorrhage [4, 5].

Recently, segmental arterial mediolysis (SAM) has been reported as a possible etiology of life-threatening abdominal, retroperitoneal, and intracranial hemorrhages [6]. SAM is a rare disease in which the intra-abdominal arterial media are segmentally dissected, resulting in the rupture of dissecting aneurysms and vascular stenosis [7–9]. It has been reported that SAM lesions are commonly found in the superior mesenteric, celiac, and renal arteries, with more than half of the patients having multiple arterial involvement [7, 10]. However, no previous reports have described the association between gallbladder hemorrhage and SAM.

Here, we present a case of gallbladder hemorrhage treated with laparoscopic cholecystectomy and the histopathological diagnosis of SAM. To the best of our knowledge, this is the first report of a gallbladder hemorrhage associated with SAM.

## Case presentation

**Fig. 1:**
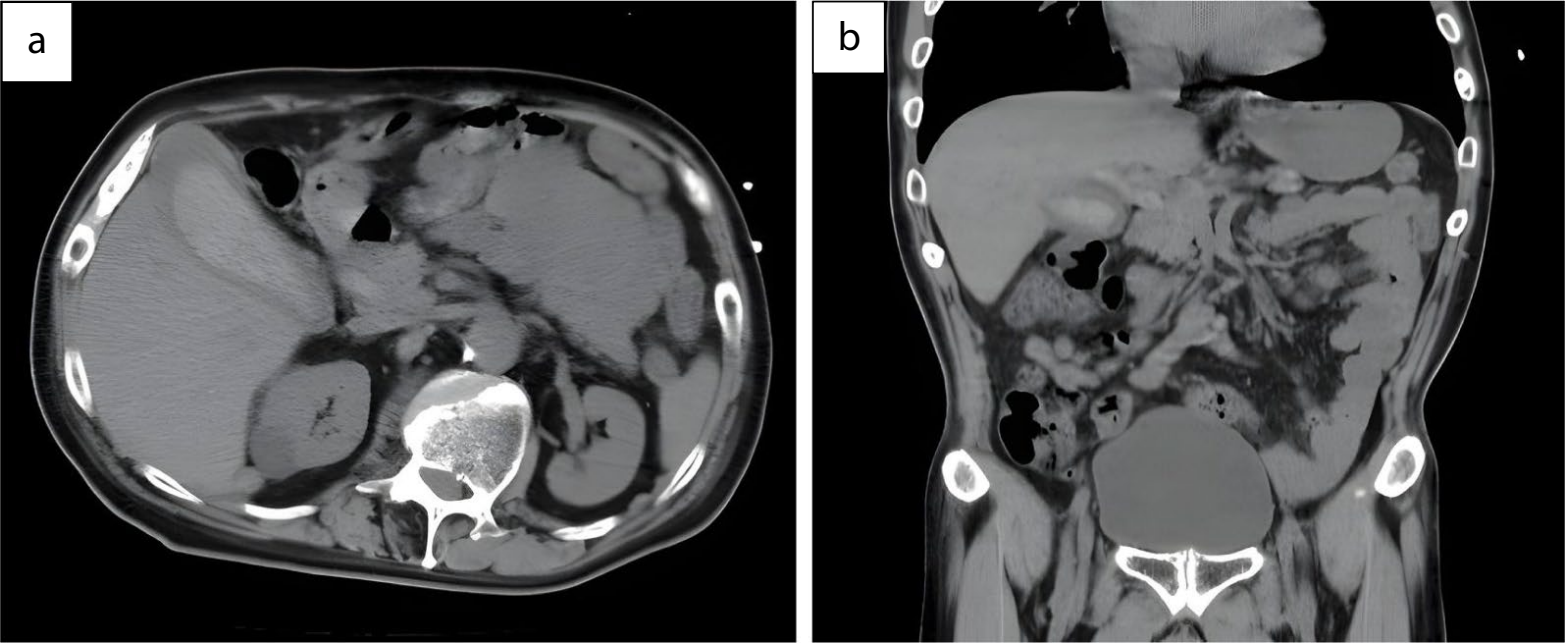
Abdominal CT images. Axial view (**a**) and coronal view (**b**) reveal diffuse thickening of the gallbladder wall and a high-density image in the gallbladder and common bile duct, suggesting gallbladder hemorrhage. CT, computed tomography

**Fig. 2:**
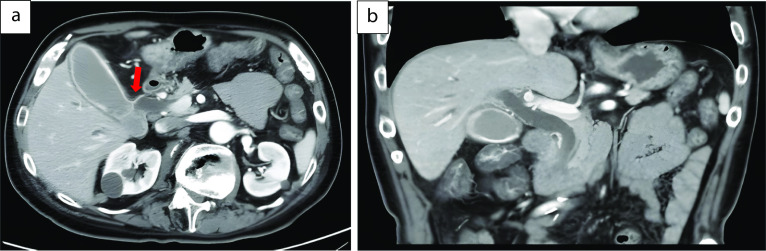
Abdominal contrast-enhanced CT. Axial view (**a**) and coronal view (**b**) reveal diffuse thickening of the gallbladder wall and dilatation of the common bile duct, but no evidence of extravasation within the gallbladder or bile duct. There are no obvious findings of gallstones, cholecystitis, tumors, or aneurysms. The red arrow indicates cystic artery. CT, computed tomography

**Fig. 3:**
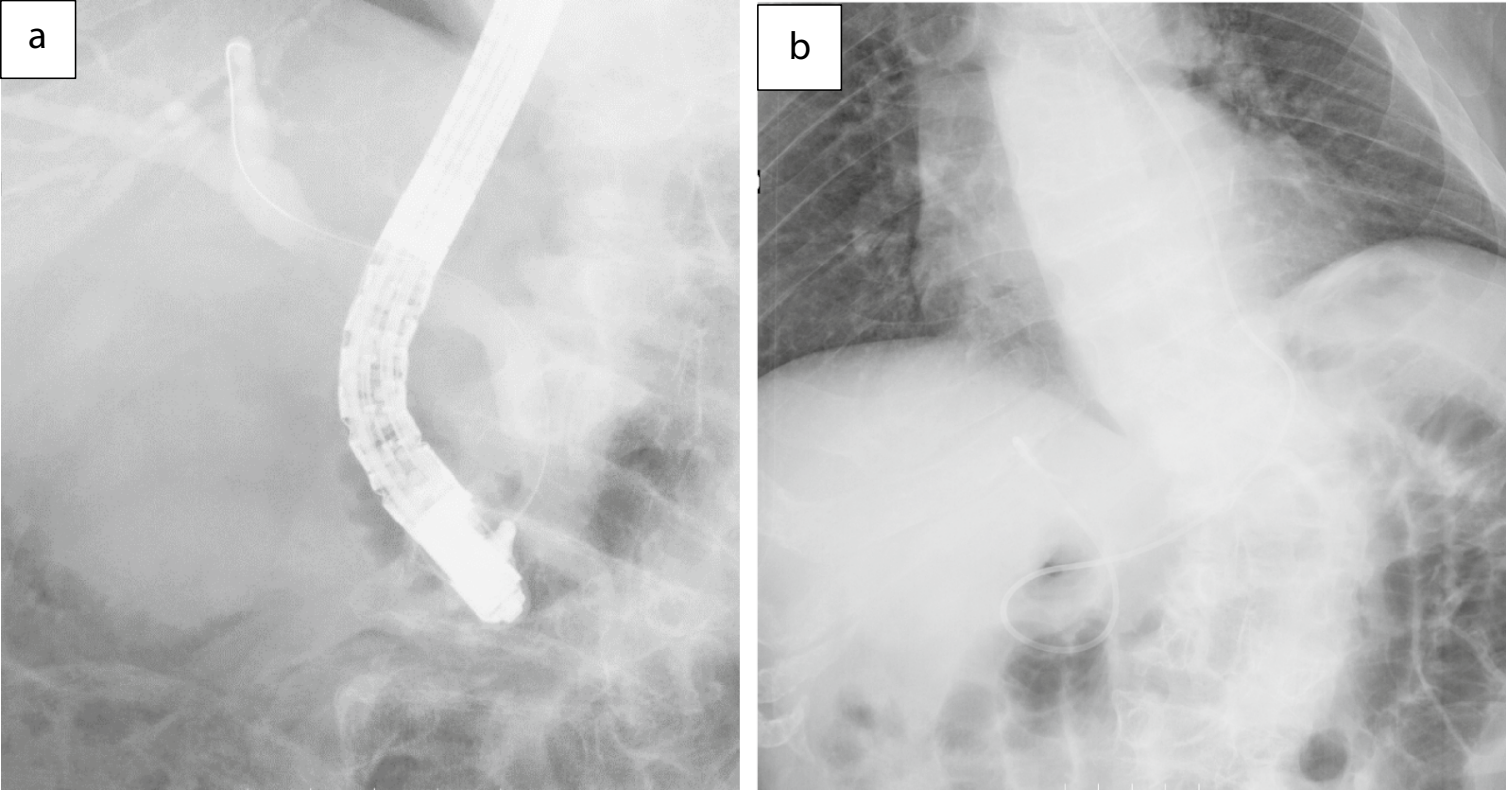
Endoscopic retrograde cholangiography images. The images reveal dilation of the bile ducts, but no obvious contrast defects in the gallbladder and bile duct (**a**). Insertion of endoscopic nasobiliary drainage tube (**b**) is shown

**Fig. 4:**
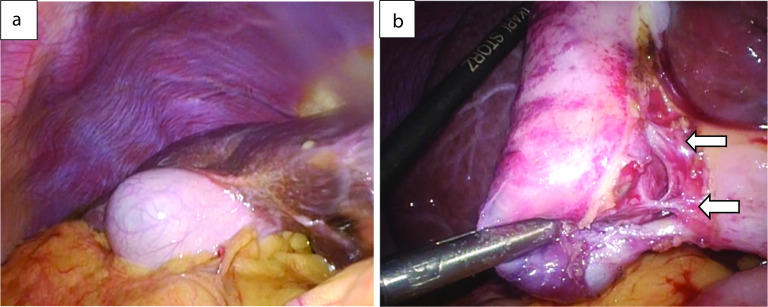
Intraoperative photographs. Intraoperatively, the gallbladder appears slightly distended, but there are no obvious signs of inflammation. White arrows indicate the cystic artery

**Fig. 5:**
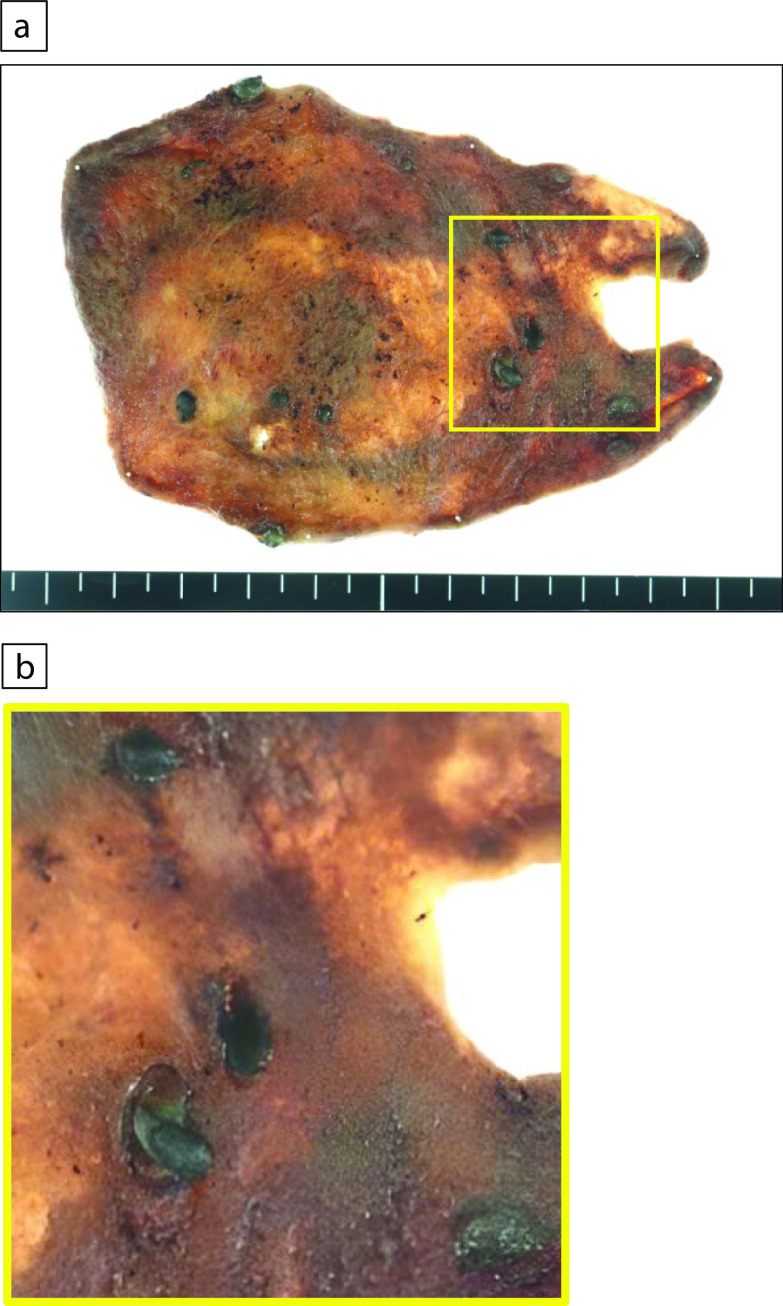
Gross appearance of the surgically resected specimen. Gross appearance of the surgically resected specimen shows 12 small lesions on the gallbladder mucosa (**a**), which are black and slightly elevated (**b**). The size of the lesions ranges from 3 to 12 mm

**Fig. 6:**
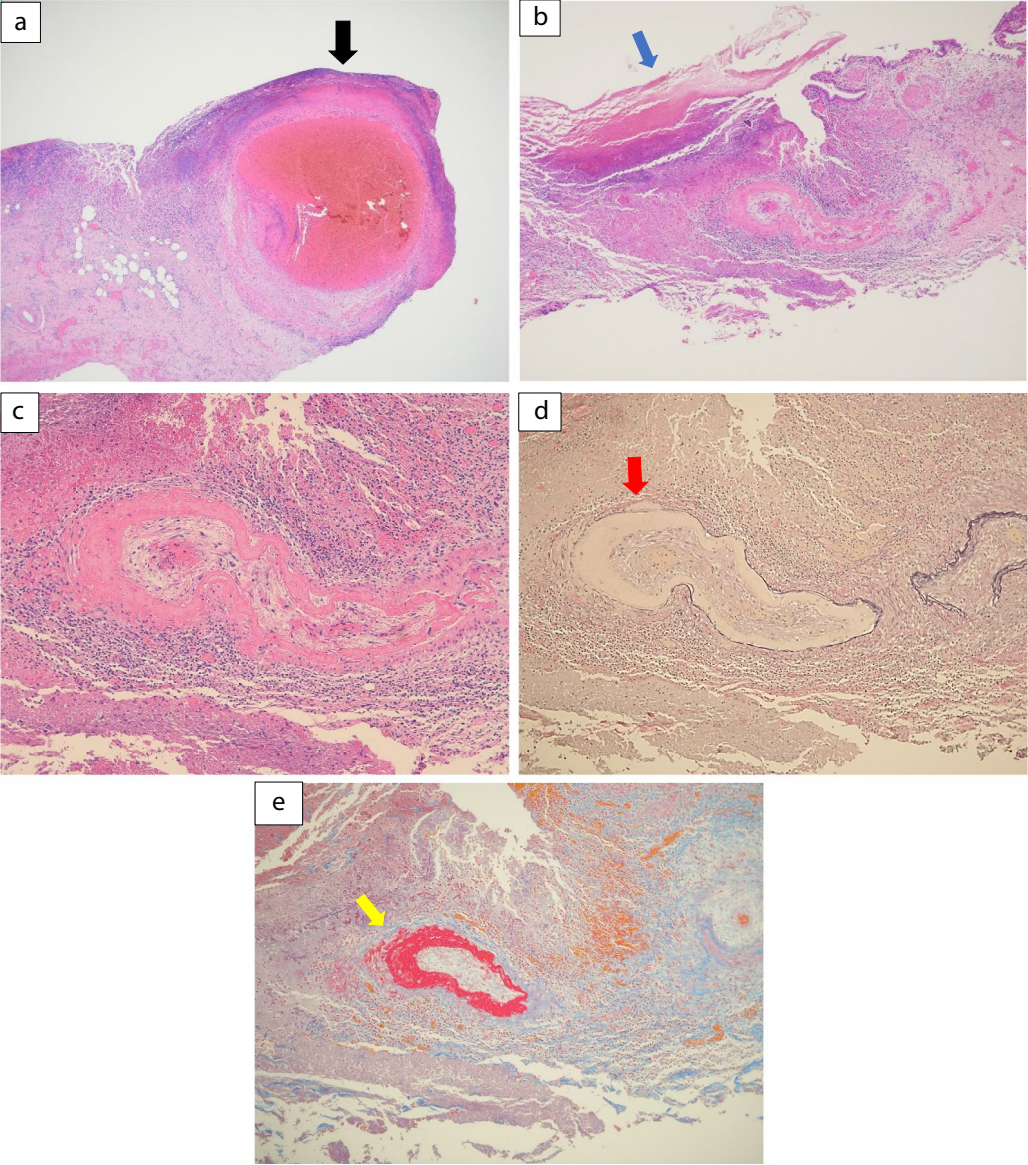
Histopathological findings. **a**, **b** Slightly elevated lesions consisting of dilated muscular arteries are seen in the gallbladder wall (black arrow) with ulceration of the overlying mucosa (blue arrow). In such lesions, focal disappearance of the internal and external elastic lamina (red arrow) and fibrinoid degeneration of the media (yellow arrow) are found in the muscular artery of the gallbladder wall (**c**–**e**). (**a**, **b** H&E stain; magnification × 40, **c** H&E stain; magnification × 100, **d** Elastica van Gieson stain × 100, and **e** Masson’s Trichrome stain × 100). H&E, hematoxylin and eosin stain

A 59-year-old man was followed-up for achondroplasia and scoliosis in our hospital’s orthopedic department. The patient was transferred to our hospital complaining of upper abdominal pain and vomiting. On admission to our hospital, his vital signs were as follows: body temperature, 36.7 ℃, blood pressure 116/77 mmHg, and pulse rate 83 beats/min. Physical examination revealed upper abdominal tenderness. Laboratory investigations revealed mild anemia (hemoglobin 10.7 g/dL) and slightly elevated inflammatory response (white blood cell count 8700/μL and C-reactive protein 2.03 mg/dL). Hepatobiliary enzymes were elevated as follows: aspartate aminotransferase (AST) 917 U/L, alanine aminotransferase (ALT) 777 U/L, alkaline phosphatase (ALP) 1411 U/L, and γ-glutamyltransferase (γ-GTP) 554 U/L. Total bilirubin (T.Bil) and direct bilirubin (D.Bil) levels were 1.2 mg/dL and 0.8 mg/dL, respectively. Computed tomography (CT) revealed diffuse thickening of the gallbladder wall and high-density images of the gallbladder and common bile duct, suggesting gallbladder hemorrhage (Fig. 1a, b). On hospital day 2, he had worsening abdominal pain, and laboratory investigations showed more elevated hepatobiliary enzymes (AST 2925 U/L, ALT 966 U/L, ALP 1252 U/L, and γ-GTP 596 U/L) and T.Bil level (2.0 mg/dL). Hb levels did not change significantly (10.8 g/dL). Contrast-enhanced CT showed diffuse thickening of the gallbladder wall, high-density imaging of the gallbladder, and dilatation of the common bile duct (13 mm), but no evidence of extravasation within the gallbladder or significant inflammation of the connective tissue surrounding the gallbladder. There were no obvious findings of gallstones, cholecystitis, tumors, or aneurysms (Fig. 2a, b). The patient was diagnosed with bile duct obstruction due to gallbladder hemorrhage. As there was no evidence of massive bleeding, and his general condition was relatively stable, we performed endoscopic nasobiliary drainage (ENBD) to reduce jaundice. During the procedure, no active bleeding from the papilla of Vater was observed, and bile discharge was confirmed. Cholangiography showed bile duct dilatation (13 mm), but no obvious contrast defect in the gallbladder or bile duct (Fig. 3a, b). The ENBD tube was successfully inserted and the procedure was completed (Fig. 3b). After the treatment, approximately 300–400 ml of bile was drained per day, and the abdominal pain was significantly relieved, and the liver enzyme and total bilirubin levels improved. There was no bleeding in drainage bag and no significant decrease in Hb levels was observed (10.2 g/dL). Therefore, we performed laparoscopic cholecystectomy on hospital day 5 to ensure hemostasis and make a definitive diagnosis, including the cause of the gallbladder hemorrhage, especially to exclude malignant lesions. Laparoscopic cholecystectomy was performed using four ports in the usual manner. Intraoperatively, the gallbladder appeared slightly distended; however, there were no obvious signs of inflammation (Fig. 4a, b). The cystic duct and artery were identified and carefully divided using the critical view of the safety technique, and cholecystectomy was completed. The gross appearance of the surgically resected specimen showed small 12 lesions on the gallbladder mucosa that were black and slightly elevated. The size of the lesions ranged from 3 to 12 mm (Fig. 5). Histopathologically, these slightly elevated lesions consisted of dilated muscular arteries in the gallbladder wall, with ulceration of the overlying mucosa. In such lesions, fibrinoid degeneration of the media and focal loss of the internal and external elastic laminae were observed in the bulging nodular muscular arteries (Fig. 6a–e). The inflammatory cell infiltration was unremarkable. Based on these findings, the histopathological diagnosis of SAM was confirmed. The patient was discharged without any postoperative complications on hospital day 12. Predischarge magnetic resonance angiography (MRA) and contrast-enhanced CT revealed no other lesions in the intracranial or abdominal visceral vessels. Serial follow-ups with CT imaging every 6 months after surgery also showed no new lesions for 3 years.

## Discussion

Biliary hemorrhage is a rare disease concept proposed by Sandblom [1] in 1948, and its frequency is estimated to be 2–5% of all upper gastrointestinal hemorrhage [3]. Of these, gallbladder hemorrhage accounts for 20–25% [1, 2]. The symptoms of gallbladder hemorrhage depend on the rate of bleeding and the presence of bile duct obstruction due to clots. Massive hemorrhage and gallbladder perforation are associated with high morbidity and mortality [11]. Therefore, early diagnosis is essential to facilitate urgent surgical management. In previously reported cases, the diagnosis of gallbladder hemorrhage was mainly made using contrast-enhanced CT, with findings of high-density fluid in the gallbladder lumen. Contrast-enhanced CT can also detect complications or underlying diseases, such as gallbladder rupture, acute cholecystitis, acute pancreatitis, and gallbladder malignancy [12–14]. Magnetic resonance imaging is reportedly useful for differentiating blood from gallstones and sludge in the gallbladder [15]. In our patient, contrast-enhanced CT showed diffuse thickening of the gallbladder wall and high-density images of the gallbladder and common bile duct, suggesting gallbladder hemorrhage. However, there were no obvious findings of gallstones, tumors, or aneurysms. As a result, we could not determine the cause of the gallbladder hemorrhage or make a preoperative diagnosis of SAM.

The treatment approach for patients with gallbladder hemorrhage remains controversial, and there are currently no guidelines for the management of this condition. Treatment decisions should be based on the patient’s clinical presentation. Endoscopic biliary drainage should be performed in patients with obstructive jaundice due to bile duct clots. If the bleeding is massive or the hemodynamic status is unstable, interventional and diagnostic angiography to embolize the offending vessel should be considered as an option for hemostasis. Previous reports have shown that transcatheter embolization (TAE) has a high success rate of hemostasis in 75–100% of patients with gallbladder and biliary hemorrhage [16, 17]. However, there is still a recognized risk of ischemic cholecystitis and exacerbation of sepsis following embolization [18]. Therefore, surgical resection would only be a rational approach in patients with gallbladder hemorrhage to ensure hemostasis and establish a definitive diagnosis, especially in patients with lesions in which malignant potential cannot be excluded. Laparoscopic surgery is preferred because it has several advantages over open surgery, including reduced bleeding and wound infection and shortened hospital stay. In our patient, there was no evidence of extravasation or massive bleeding on preoperative CT, and the patient’s general condition was stable. Therefore, we planned to perform laparoscopic cholecystectomy to ensure hemostasis and establish a definitive diagnosis, including the cause of the gallbladder hemorrhage after biliary decompression.

SAM was originally described by Gruenwald in 1949 as coronary artery disease in newborns [19]. Slavin et al. histologically identified the characteristic features of segmented and island-like residual media in aneurysms in the abdomen and proposed the term “segmental mediolytic arteritis” in 1976 [20]. Subsequently, it became apparent that SAM represents a different condition from typical intra-abdominal aneurysm characterized by atherosclerotic changes or arteritis, leading to its renaming as “segmental arterial mediolysis” in 1995 [21]. As outlined by Slavin et al., the diagnostic lesions of SAM include mediolysis, separation, arterial gaps, and reparative fibrosis. Mediolysis refers to partial or total vacuolization and lysis of the outer arterial media. This leads to the weakening of the outer layer of the media, formation of arterial gaps, patchy transmural loss of the external elastic lamina, and separation of the media from the adventitia. Ultimately, dissecting hematomas and aneurysms occur at the arterial gaps, which can result in sudden massive intra-abdominal or retroperitoneal hemorrhage.

SAM most commonly affects the abdominal aortic branches, which are considered medium-sized vessels. In a review by Nedaa et al. [7] involving 143 cases, the most common site of SAM lesions was the superior mesenteric artery (SMA) (53.1%), followed by the hepatic (44.8%), celiac (35.7%), renal (25.9%), and splenic (24.5%) arteries. More than half of the patients (62.2%) showed involvement of multiple arteries. Naidu et al. [10], based on 111 cases, reported that the renal arteries (47%) were the most frequently affected, followed by the SMA (47%) and celiac (46%), hepatic (23%), iliac (18%), and splenic (24.5%) arteries. Most patients (57%) had the involvement of two or more vascular territories. However, neither report has mentioned cases of SAM involving the cystic artery or its branches.

Although histopathological examination is the gold standard for the definitive diagnosis of SAM, it requires an invasive arterial biopsy, which is often not feasible and is not always available. Therefore, the diagnosis of SAM is supported by the characteristic patterns of imaging findings, such as CT angiography, MRA, and catheter angiography, and by the clinical and laboratory exclusion of other differentials. Slavin et al. [22] listed several imaging features of SAM, including arterial dilatation, single aneurysm, multiple aneurysms creating a string-of-beads image, dissection, arterial stenosis, and arterial occlusion. In our patient, preoperative contrast-enhanced CT imaging showed diffuse thickening of the gallbladder wall and a high-density image of the gallbladder, but no imaging features of SAM, as described above. The 12 slightly elevated lesions in the gallbladder mucosa of the surgically resected specimen consisted of dilated muscular arteries with fibrinoid degeneration of the media, focal loss of the internal and external elastic lamina in the gallbladder wall, and ulceration of the overlying mucosa. Histopathological findings suggested that rupture of such muscular arteries may have caused the gallbladder hemorrhage. However, these slightly elevated gallbladder lesions were too small to be detected using diagnostic imaging modalities.

It has been reported that SAM treatment should be tailored to individual clinical presentations, although there are no formal guidelines for its management [8, 23]. Previously reported cases of aneurysms and dissections were initially managed conservatively by monitoring lesion progression, with a relatively low morbidity rate [6, 7, 24]. However, cases of massive hemorrhage, bowel infarction, or arterial rupture require endovascular and/or surgical treatment. A systematic review of 143 cases reported by Skeik et al. [7] showed that TAE was the most common treatment modality (27.9%) followed by abdominal organ surgery (23.5%). Regarding the prognosis of SAM, the reported mortality rates range from 7 to 40% due to acute intra-abdominal hemorrhage [6, 10]. Although SAM is a potentially life-threatening condition, most patients stabilize or regress if they survive the acute phase. However, a follow-up imaging study by Naidu et al. [10] (median follow-up, 12 months) showed that 19 (20%) of 97 patients with SAM showed disease progression or new lesions. In addition, approximately 30% of patients with SAM have multiple simultaneous or metachronous aneurysms [23]. Given the specific and spatiotemporal characteristics of SAM, they recommended serial follow-ups with CT imaging after the initial diagnosis to rule out disease progression or new lesions. In the present case, a definitive diagnosis of SAM was made by histopathological examination of a surgically resected specimen. However, a definitive diagnosis of SAM, even postoperatively, is important because it allows for the recognition of the possibility of simultaneous and/or metachronous multiple lesions and the potential for progression. In our patient, after the definitive diagnosis of SAM, pre-discharge MRA and CT imaging were performed, as well as serial postoperative follow-ups with CT imaging every 6 months for 3 years, and we confirmed no evidence of disease progression or new lesions. Therefore, we believe that surgery would have been valuable in our case, not only for hemostasis, but also for a definitive diagnosis of SAM.

Shenouda et al. [25] suggested that the true prevalence of SAM is likely to be underestimated in the literature, as the diagnosis of SAM, including histopathological diagnosis, may be missed because of the limited awareness resulting from the rarity of SAM. In fact, Inada et al. [26] reported 27 cases of unrecognized SAM by histopathological review of cases of ruptured visceral arterial aneurysms. Our case report shows that SAM can cause gallbladder hemorrhage, although we could not diagnose SAM preoperatively. Therefore, considering the data from previous reports and our case, SAM should be considered in the differential diagnosis of intra-abdominal hemorrhages, including gallbladder hemorrhages, even in cases without the typical clinical and imaging features of SAM.

## Conclusion

Here, we report the first case of gallbladder hemorrhage associated with SAM. This case report shows that SAM can cause gallbladder hemorrhage. Therefore, SAM should be considered in the differential diagnosis of gallbladder hemorrhage.

## Data Availability

The authors declare that all the data are available within this article.

## Abbreviations

SAM: Segmental arterial mediolysis
CT: Computed tomography
TAE: Transcatheter embolization
MRA: Magnetic resonance angiography
SMA: Superior mesenteric artery
